# Assessing impact of agroecological interventions in Niger through remotely sensed changes in vegetation

**DOI:** 10.1038/s41598-022-27242-3

**Published:** 2023-01-07

**Authors:** Vikalp Mishra, Ashutosh S. Limaye, Federico Doehnert, Raffaella Policastro, Djibril Hassan, Marie Therese Yaba Ndiaye, Nicole Van Abel, Kiersten Johnson, Joseph Grange, Kevin Coffey, Arif Rashid

**Affiliations:** 1 Earth System Science Center, The University of Alabama in Huntsville, 320 Sparkman Dr., Huntsville, AL USA; 2 NASA-SERVIR Science Coordination Office, Marshall Space Flight Center, Huntsville, AL USA; 3 NASA Earth Science Branch, Marshall Space Flight Center, Huntsville, AL USA; 4 World Food Programme Regional Bureau for Western Africa, Dakar, Senegal; 5 World Food Programme Niger Country Office, Niamey, Niger; 6 Bureau for Humanitarian Assistance, United States Agency for International Development (USAID), Washington DC, USA

**Keywords:** Environmental impact, Climate-change mitigation

## Abstract

Water scarcity is a major challenge in the Sahel region of West Africa. Water scarcity in combination with prevalent soil degradation has severely reduced the land productivity in the region. The decrease in resiliency of food security systems of marginalized population has huge societal implications which often leads to mass migrations and conflicts. The U.S. Agency for International Development (USAID) and development organizations have made major investments in the Sahel to improve resilience through land rehabilitation activities in recent years. To help restore degraded lands at the farm level, the World Food Programme (WFP) with assistance from USAID’s Bureau for Humanitarian Assistance supported the construction of water and soil retention structures called half-moons. The vegetation growing in the half-moons is vitally important to increase agricultural productivity and feed animals, a critical element of sustainable food security in the region. This paper investigates the effectiveness of interventions at 18 WFP sites in southern Niger using vegetative greenness observations from the Landsat 7 satellite. The pre - and post-intervention analysis shows that vegetation greenness after the half-moon intervention was nearly 50% higher than in the pre-intervention years. The vegetation in the intervened area was more than 25% greener than the nearby control area. Together, the results indicate that the half-moons are effective adaptations to the traditional land management systems to increase agricultural production in arid ecosystems, which is evident through improved vegetation conditions in southern Niger. The analysis shows that the improvement brought by the interventions continue to provide the benefits. Continued application of these adaptation techniques on a larger scale will increase agricultural production and build resilience to drought for subsistence farmers in West Africa. Quantifiable increase in efficacy of local-scale land and water management techniques, and the resulting jump in large-scale investments to scale similar efforts will help farmers enhance their resiliency in a sustainable manner will lead to a reduction in food security shortages.

## Introduction

Since the droughts in the 1970s and early 80s, land degradation and water scarcity are major challenges in the Sahel region of West Africa. Dry and arid lands are even more vulnerable to land degradation and eventual desertification^1,2^. Low annual rainfall combined with a short and distinct rainy season and overuse (such as overgrazing), makes land and vegetation susceptible to drought and degradation^3^. Sustained water availability is vital for vegetative growth while arresting the land degradation from erosion, nutrient loss etc.^4^. Climate and human activities have resulted in a significant increase in land degradation, which in turn has a significant impact on crop production and food security^5^, hydrology^6^ and in some cases even results in conflicts^7^. Soil conditions can be improved through targeted local-scale land management practices and increase their resilience to drought^8,9^. Typical land rehabilitation includes either planting drought-resistant plant species or through soil water conservation (SWC) mechanisms^10–13^.

Several efforts have been made towards land restoration through SWC interventions^14–16^. In the Sahel region of West Africa, farmers have used an array of land management techniques for land rehabilitation including zai, half-moons, and stone bunds^17–24^. Half-moons, a small semi-circular pond, in particular have been used in the Sahel region for several decades to restore degraded lands^25,26^. The half-moons stores rainwater and provides moisture over an extended period to the vegetation grown in the excavated area and its vicinity^27^. The half-moons are critical during the rainy season because these can not only provide additional moisture to the vegetation during the dry periods^28^ but can also limit surface runoff. The half-moons help with water conservation, reduce soil erosion, and help retain soil nutrients^18,29^.

Multiple organizations including the World Food Programme (WFP) serve as critical links between donors and local agrarian communities. Farmers, with the support of these organizations, have been involved in SWC projects in the Sahel region to improve soil fertility, control runoff, and restore degraded natural ecosystems^25^. For the past several decades, both WFP and U.S. Agency for International Development (USAID) have made major investments in the Sahel to improve resilience through land rehabilitation activities that improve water conservation and enhance food crops and fodder production in previously barren lands. These activities were carried out at large scales throughout southern Niger as part of USAID’s Bureau of Humanitarian Assistance Food Security Initiatives. WFP has meticulously compiled a comprehensive record of interventions in southern Niger and this study outlines a methodology to evaluate the efficacy of those interventions. It is hypothesized that the additional moisture retention by the half-moons will substantially improve vegetation growth, which can be captured in satellite measurements of greenness. A better understanding of the impact of various agricultural interventions will improve the cost efficiency of community planning in low-resource environments.

An increase in soil moisture directly leading to enhanced agricultural production has been reported at several sites^27,30–32^. However, a complete picture of intervention impacts at a wider scale is challenging due to limited access to some of the remote locations, security concerns, and costs of data collection, capacity and resource availability^8,33–36^. Furthermore, often times the baseline household and agricultural survey data were not gathered prior to the implementation of the intervention, posing a challenge to assessing the effectiveness of these interventions at the later stages^11^. Several earlier studies have raised the issue of alternate methods to mitigate some of the issues concerning ground data collections and for standardizing the evaluation efforts^37–40^.

One alternative to costly and time-consuming ground surveys is the use of satellite remote sensing data for impact analysis^9,11,19,41–44^. Remote sensing methods can complement other assessment methods by considering data at a landscape scale, supporting historical analysis, and producing meaningful quantitative metrics. Satellite-driven vegetative greenness conditions can be used as a proxy for improved soil and water conditions to quantify the intervention impact^1,9,11,45^. Normalized Difference Vegetation Index [NDVI^46,47^] can measure the vegetation greenness and can quantify the nature and intensity of vegetation change across space and time over large spatial scales^48^. With the availability of long historical records, remotely sensed vegetation condition information can provide valuable insight into greenness before and after interventions, therefore avoiding the need for a baseline ground survey. Furthermore, satellite data processing can be automated therefore can reduce the risks of potential human bias in the survey and interpretation of results^49^. The aim of this study was to assess the applicability of satellite-based observations in quantifying the SWC intervention impact over small plots in southwestern Niger. In this case study, we analyzed multi-year NDVI data (2010-2019) from Landsat 7 to assess the impact of land rehabilitation activities on agricultural production and resilience to drought. We envision similar analyses will provide concrete, independent foundations for a more sustained use of local-scale land restoration methodologies, that will serve as an important bridge to increasing food security in the most vulnerable populations around the world.

## Results

### Pre- and post-intervention analysis

**Figure 1:**
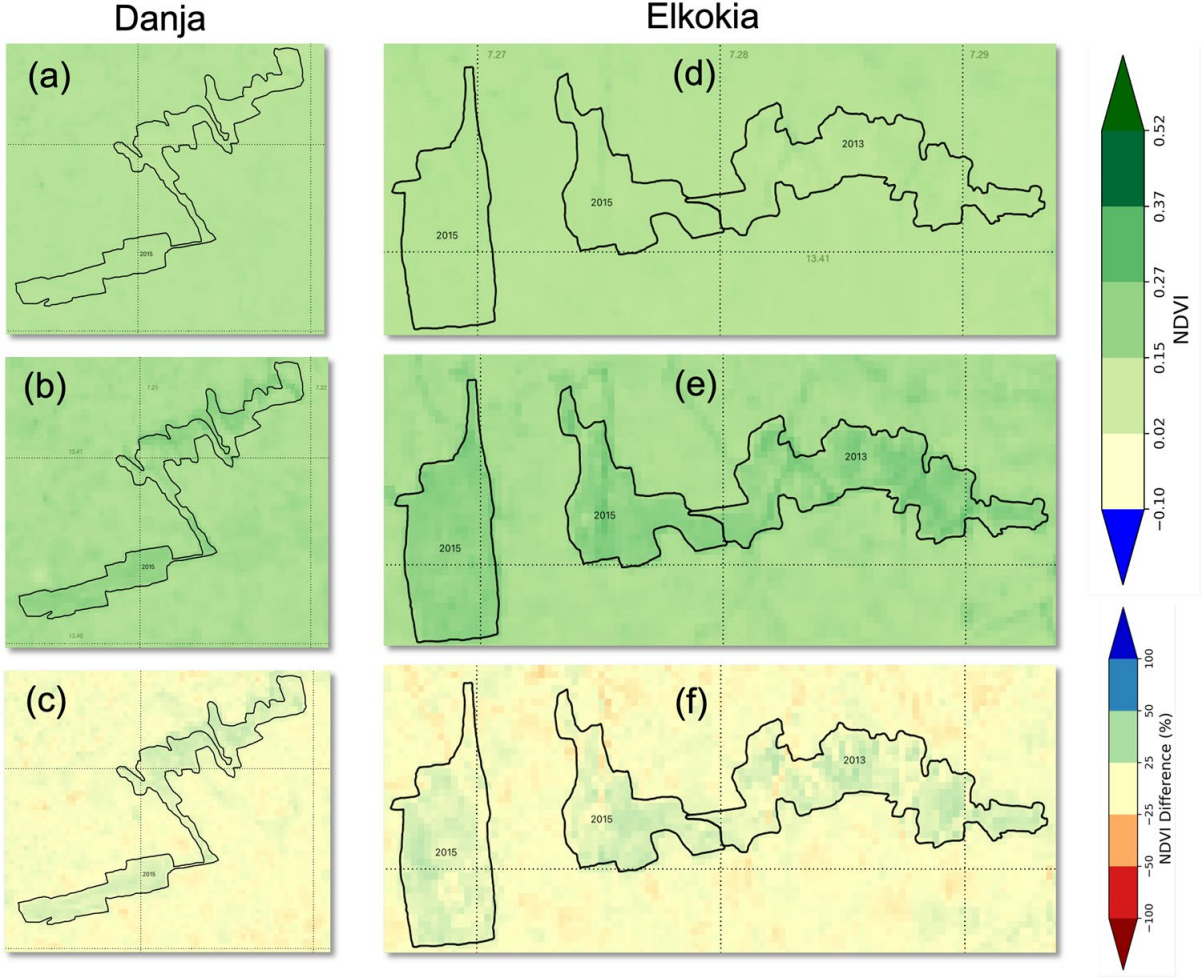
NDVI map for two locations Danja (**a**-**c**) and Elkokia (**d**-**f**) for the month of Jun before [year 2013 (**a**) and (**d**)] and after [year 2019 (**b**) and (**e**)] interventions. The bottom panel (**c** and **f**) shows the percent difference in NDVI values from Jun 2013 to Jun 2019.

Figure 1 shows the NDVI map for for the month of June 2013 and 2019 at two of the locations (Danja and Elkokia). These sites were intervened from 2013 to 2015, and the fig 1. The figure clearly shows that ares surrounding the intervention sites also showed a modest increase in NDVI values (5%), the intervened sites showed significantly higher NDVI values post-intervention (>25%) in 2019 when compared to 2013 period.

**Figure 2:**
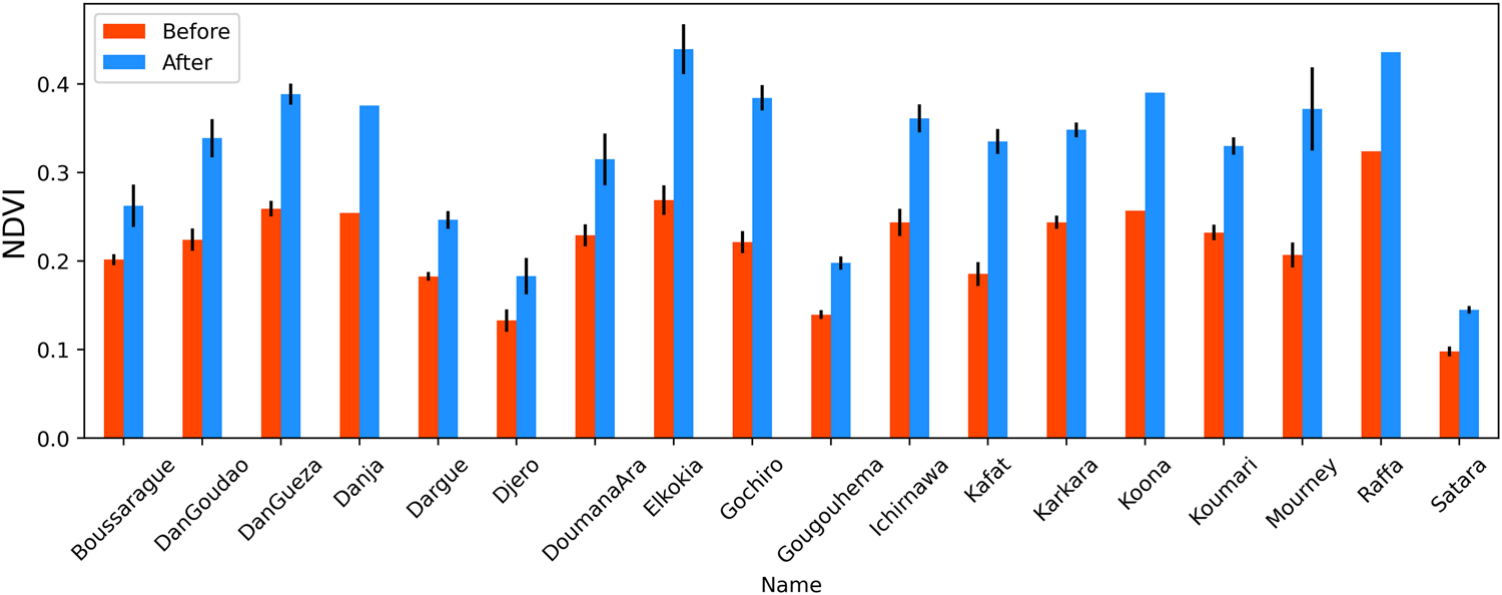
Mean peak NDVI across all sub-polygons for all sites intervened before and after intervention. The error bars represent the standard error in mean NDVI values from different sub-polygons within a site. [Danja, Koona and Raffa are single polygon sites, hence no error bars].

The peak NDVI value during the peak growing season (Aug–Oct) for each of the sites showed a significant improvement post-intervention as seen by Landsat 7 (Fig. 2). On average, the peak NDVI value increased by 49.7% (from 0.217 to 0.325) across all sites after the interventions (Table 1). NDVI increased at four sites by more than 60%, with the maximum enhancement of 81.1% observed for Kafat (NDVI increased from 0.185 to 0.335). We observed the smallest NDVI increase of 29.7% (from 0.202 to 0.262) for Boussarague; however, the difference in NDVI is also positive and statistically significant using two-tailed t-test (*p*<0.001).

The rainfall during months preceding the peak growing season is expected to have a significant impact on vegetation conditions. Therefore, in this analysis, we considered the total rainfall data for two months prior to the peak growing season. Table 1 shows the annual peak NDVI values (Aug–Oct) before and after the interventions for each of the sites, in addition to the average total rainfall from two months before the peak NDVI season June to August. The table shows that the total rainfall post-intervention is higher (12.3% on average) for all sites, which can be attributed to the above-average precipitation in recent years (2018–2020). Although rainfall is one of the primary drivers of vegetative growth, the table clearly indicates that the change in peak NDVI values is not linearly related to the differences in total rainfall before and after the intervention. Even though the rainfall increased by 12%, the NDVI jumped by nearly 50% at these sites after the intervention, as compared to the vegetation before the intervention.

**Table 1:** Summary of mean annual peak NDVI values (Aug–Oct) before and after intervention with the mean total rainfall (Jun–Aug) from 2010 to 2020.

Site	NDVI		Rainfall (mm)		Difference (%)	
Name	Before	After	Before	After	NDVI	Rainfall
Boussarague	0.202	0.262	347	383	29.7	10.3
Dan Goudao	0.224	0.339	348	392	51.3	12.6
Dan Gueza	0.259	0.388	310	331	49.8	06.7
Danja	0.254	0.375	407	410	47.6	00.7
Dargue	0.183	0.246	330	380	34.4	15.1
Djero	0.133	0.183	372	424	37.6	14.0
Doumana Ara	0.229	0.315	354	417	37.6	17.8
Elkokia	0.269	0.439	395	386	63.2	02.2
Gochiro	0.221	0.384	356	424	73.8	18.9
Gougouhema	0.140	0.198	274	312	41.4	14.0
Ichirnawa	0.244	0.361	388	428	48.0	10.3
Kafat	0.185	0.335	266	325	81.1	22.4
Karkara	0.244	0.348	336	352	42.6	04.9
Koona	0.257	0.390	359	422	51.8	17.5
Koumari	0.232	0.330	275	311	42.2	13.3
Mourney	0.207	0.372	360	409	79.7	13.6
Raffa	0.324	0.436	360	419	34.6	16.4
Satara	0.098	0.145	279	311	48.0	11.2
Mean	0.217	0.325	340	380	49.7	12.3

**Figure 3:**
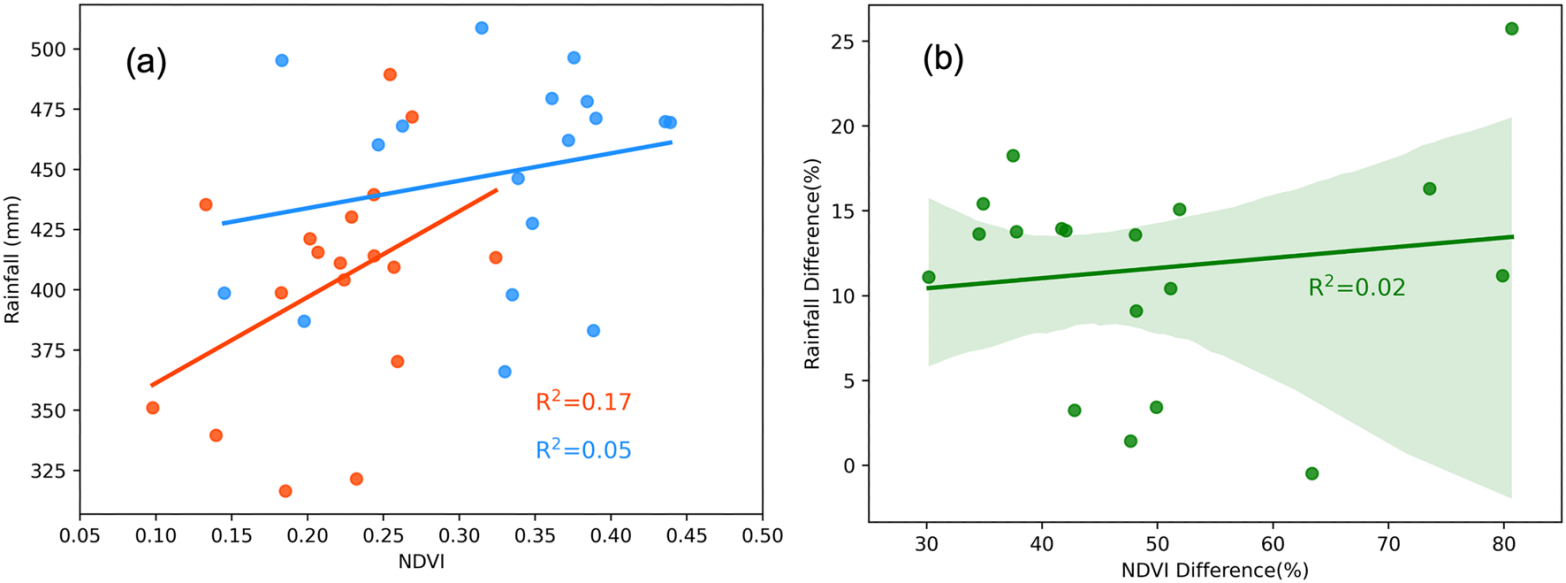
Scatter plots (**a**) showing relationship between the mean peak NDVI and total rainfall before (red) and after the intervention (blue) and (**b**) scatter plot between percent differences in NDVI and rainfall before and after intervention for each of the sites.

Figure 3(a) shows the scatter plot between the peak NDVI and total rainfall before and after the intervention. The results show that there was some, although weak, relationship between the NDVI and rainfall before the intervention ($R^2$ = 0.24). Interestingly, the relationship between the two variables was reduced by 36% ($R^2$ = 0.15) after the intervention. This confirms the assumption that there are factors other than total rainfall that contributed to the vegetative growth in the region and that these external factors seem to play an even greater role post-intervention period, with the construction of half-moons being the obvious change between the pre-and post-intervention NDVI estimates. Despite above-normal rainfall during the later stages of the study years (2018–2020, Fig. 9a), the scatter plot (Fig. 3b) between the percent difference in total rainfall and NDVI peak shows that there is no linear relationship between these two variables. The analysis indicates that the impact of increased rainfall in increasing vegetative growth is negligible after the interventions.

**Figure 4:**
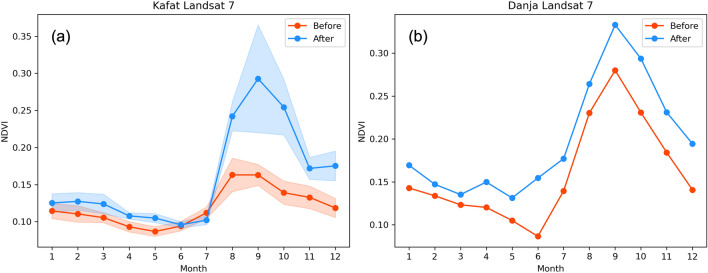
Average monthly NDVI values for two sites, Kafat and Danja, from Landsat 7 before and after the intervention. The shaded areas on the left side panels represent one standard deviation computed from multiple polygons at each site.

Examining the monthly NDVI values give insights into the natural vegetative growth cycle before and after the intervention. For instance, monthly NDVI values across Kafat and Danja are shown in Fig. 4. The shaded portion represents the standard deviation of mean NDVI values across multiple polygons at the same site (4 in the case of Kafat). WFP built half-moons in two of the sites in 2018 and the rest in 2019. Figures show that during the months of January–July (pre-rainy season), NDVI values before and after interventions were similar. However, for months during and after the rainfall season, the mean monthly NDVI values begin to deviate. The post-intervention NDVI values (blue lines in Fig. 4) are substantially higher than pre-intervention (red lines in Fig. 4) from August through December. The increase is consistent across all polygons as evident by the standard deviations. The Danja site has only one polygon ($\sim$42 ha) that was developed in 2015. Danja allows us to analyze and compare a few years of data both before and after interventions. Moreover, the post-intervention period contains at least three years of below-normal rainfall, with 2017 as one of the most significant rainfall deficit years (deficit of more than 100 mm from the long-term average), thus providing a good mix of rainfall distributions to assess the impact of interventions using NDVI. Similarly, the mean monthly NDVI values were close (although post-intervention values were consistently slightly higher) in the relatively dry months. The difference became even more pronounced during the wet months, with the mean peak rising from 0.27 to 0.33.

All intervention sites showed similar trends where NDVI values after intervention were consistently higher than the NDVI values before intervention. This higher difference in NDVI value is also observed for a couple of months after the rainy season, indicating prolonged greenness. Overall, for dry months the NDVI differences before and after interventions were in the range of 0.015–0.03, which more than doubled to 0.044–0.063 during the months when NDVI values peaked. When analyzed together, Table 1 and Figure 4 show that the half-moons at all sites were associated with a statistically significant 49.7% increase in the peak vegetation as compared to the pre-intervention period, even after accounting for differences in rainfall during the time periods.

### Control analysis

During the planning and implementation of these interventions, no control site was specifically identified. Therefore, for this analysis, we have taken an indirect approach where a pair of polygons from the same site that were developed a few years apart were used as an experiment (or intervened) and control sites. This approach ensured that the later-intervened area was suitable for the intervention, and hence is an appropriate comparator. A total of 7 pairs (Table 2) across multiple sites were found that were used for control analysis.

**Table 2:** Table showing the potential control and experiment sites (Pastoral/Agricole) with their coverage (ha) along with the mean NDVI values for baseline and experimental phase. [$^*$Agricole site].

Site	Intervention			Control			NDVI (Baseline)		NDVI (Experiment)	
Name	ID	Year	Area	ID	Year	Area	Control	Exp.	Control	Exp.
Dan Goudau	1	2013	370	0	2018	110	0.118	0.121	0.122	0.154
Dan Gueza$^*$	0	2015	310	6	2018	50	0.130	0.148	0.149	0.180
Dargue	4	2016	90	3	2019	11	0.124	0.127	0.149	0.176
Djero	2	2013	17	1	2017	14	0.101	0.118	0.088	0.157
Gougouhema	1	2014	67	11	2019	30	0.100	0.094	0.113	0.121
Karkara	0	2014	59	9	2018	15	0.122	0.110	0.143	0.177
Satara	2	2013	13	4	2019	47	0.065	0.060	0.087	0.105
Mean							0.109	0.111	0.122	0.153

**Figure 5:**
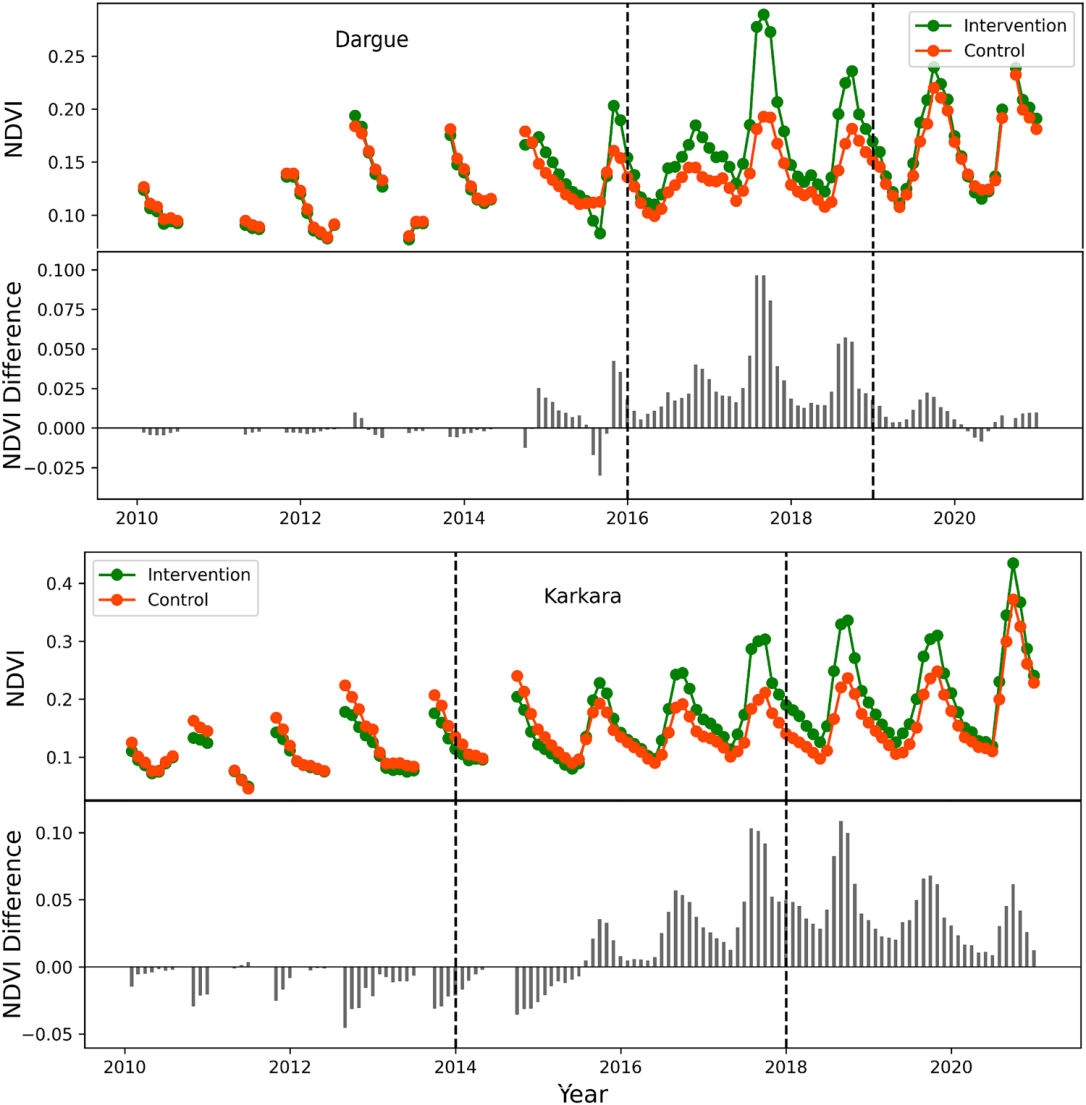
NDVI values at the control and intervention sites from 2010 to 2020 for Dargue and Karkara. The vertical lines show the beginning and end of control/experiment periods.

Figure 5 shows the NDVI time series for Dargue and Karkara at the intervention and control sites. The baseline NDVI values for both intervention and control sites were similar or in some cases less than the control sites. During the experiment period, as hypothesized, the NDVI values in the intervention site significantly increased compared to the control sites. After the end of the experiment period (when both sites were developed), the NDVI values of the control sites began to match the NDVI values from the intervention site due to the increased greenness. The analysis shows that interventions have a clear positive impact on vegetative greenness in the region. The analysis shows that the NDVI values during the baseline period for both experimental and control sites were similar. The *p*-value was found to be 0.4, therefore, the null hypothesis (that the means are statistically similar) cannot be rejected. However, during the experiment period, the average difference in mean NDVI between the control and experimental site was statistically significant (0.028) with a *p*<0.05. The increase in vegetation brought by the interventions sustained till date, which illustrates the sustainability of the interventions to provide the positive results for years to come. That is particularly notable, given the increasing threat of erratic rainfall brought by climate change.

**Figure 6:**
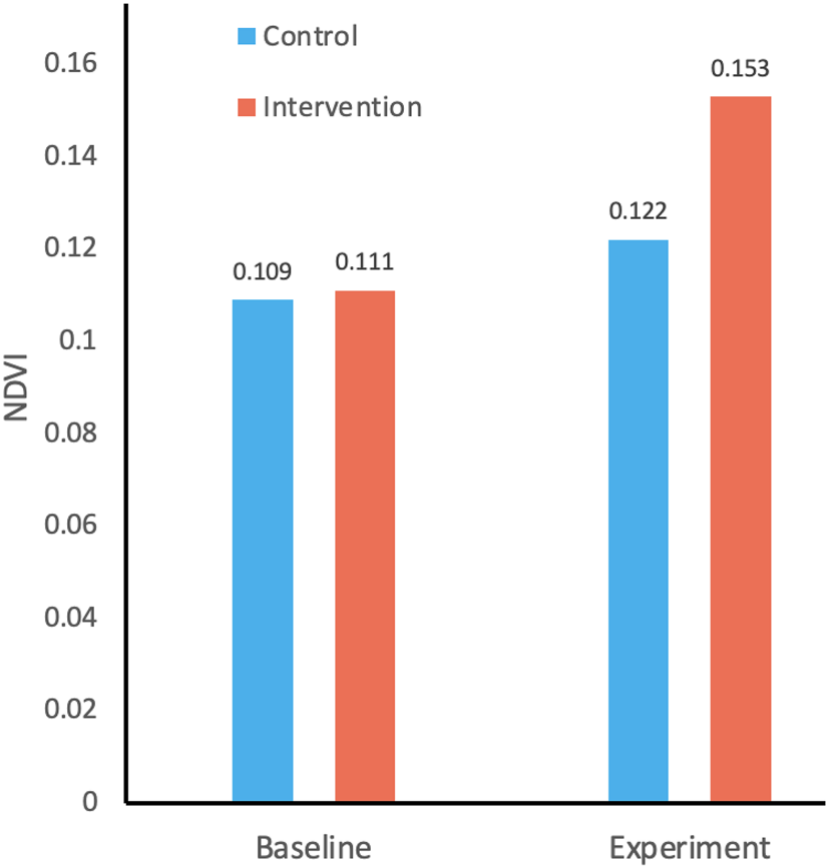
Difference in mean NDVI during the baseline and experiment period for control and intervened sites.

Overall, the NDVI analysis shows that the vegetation during the baseline period was comparable between the two sets of polygons. During the experiment period, the vegetation increased more than 25% in the polygons with half-moon interventions compared to control polygons (Fig. 6).

### BACI analysis

We performed the Before-After Control-Impact (BACI)^50,51^ analysis to further quantify the impact of interventions during the experiment period.^51^ suggested a random sampling of control and experiment sites over space and time. However, due to limited number of sample pairs, we used a randomized bootstrap (with 50 iterations) to select a combinations of the control and experiment site with varied area and intervention periods as proxy of true random sampling model design. The analysis shows that there was a BACI contrast of −0.061 with nearly 56% of relative contrast in NDVI value. The negative value of BACI contrast (in the units of NDVI) indicates that greenness has increased in the experimental site with respect to the control site, relative to NDVI values during the baseline period. The relative contrast (a ratio of BACI contrast to mean baseline NDVI from the experiment site) is a unitless normalized value used to express the impact of intervention as a percentage.

## Discussion

The satellite-derived vegetative greenness can be used to assess the impact of intervention activities. However, certain limitations must be considered when interpreting the results. In this preliminary study, we assume that precipitation, and thus the available moisture content in the soil, play the most prominent role in natural vegetative growth (most of the restoration sites being pastoral) and can explain some of the variances in annual vegetative growth conditions. It must be noted that soil moisture (or precipitation) alone may not be the only influencing factor. Further, the satellite data used in this analysis are based on visible and near-infrared bands that cannot penetrate through clouds. Therefore, under cloudy conditions, there could be significant data gaps. We mitigated this challenge by using Landsat data from 2010 only, ensuring data availability of more than 74% for months when NDVI values peaked. In this analysis, we analyzed relatively larger-sized polygons, however, Landsat’s spatial resolution of 30 m may not be appropriate for smaller (<1 ha, approx.) intervention sites. The use of harmonized multi-sensor data products including more recent Landsat 8 and 9 data along with Sentinel-2 data has the potential to mitigate some of these limitations^52,53^. The inclusion of data from cloud-penetrating synthetic aperture radar (SAR) onboard Sentinel-1 can further minimize data gaps.

Another gap in this analysis is testing the robustness of the interventions under drought conditions. For most of the sites, the rainfall during the post-intervention was higher than the long-term mean. Therefore, we could not assess the efficacy of the interventions under drought conditions. Continuous monitoring of these sites for longer periods of time will give better insight into the effectiveness of the interventions in a sustainable manner.

The results from this case study clearly shows that satellite observations can be used for impact analysis while addressing some of the challenges laid out by^8,11^ and others. Although, this study focused on the vegetative conditions as a proxy of intervention impact assessment, other satellite derived environmental variables such as the evapotranspiration^54–56^, soil moisture^57,58^ etc. can also provide critical information related to the interventions, however such observations are derived using either thermal or microwave bands and therefore are of relatively coarser resolution than visible band driven NDVI. Several attempts have been made to downscale the coarser scale soil moisture^59,60^ and evapotranspiration^61,62^ to field scales that can be utilized for such applications.

This analysis covers 18 sites, and multiple polygons at each site, in southern Niger where WFP assisted to develop half-moons between 2013 and 2020 using a pre/post assessment approach. The satellite-based NDVI measurements were used to assess the impact of the half-moons on vegetative conditions. Using Landsat 7 imagery, our analysis showed a statistically significant increase in the peak NDVI values of nearly 50% after the half-moons were constructed compared to pre-intervention years, an indication of improved grazing land for pastoralists and cropland for farmers. Analysis of vegetation at a smaller set of 7 intervention sites and nearby control sites suggests that the interventions had a significant impact on NDVI values, whereas the control sites showed modest improvement in vegetation conditions. An increase in vegetation greenness of more than 25% was found at the intervention sites, as compared to the control sites. Overall, the analysis shows that the half-moons contribute to a substantial improvement in the greenness of landscapes. Additional work is needed to link the increased greenness to crop productivity analysis; however, these results provide actionable evidence to support scaling up half-moon interventions as an effective land management practice to increase agricultural production in arid ecosystems and build resilience to drought for subsistence farmers.

## Methods

### Study area

**Figure 7:**
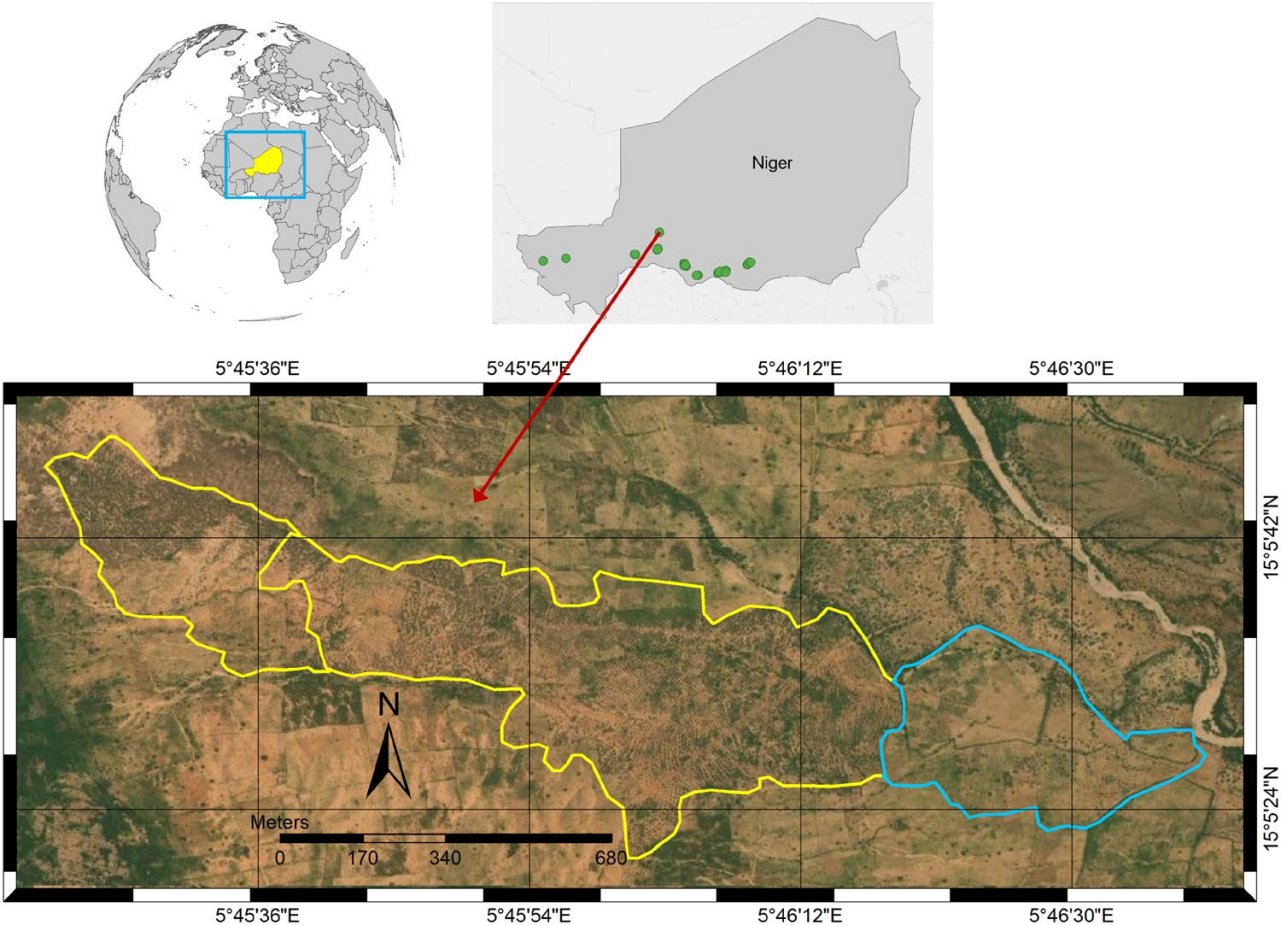
WFP intervention sites in southern Niger include interventions intended for agricultural (blue) and pastoral (yellow) uses. The figure was created using ArcGIS Pro 3 (www.arcgis.come).

**Figure 8:**
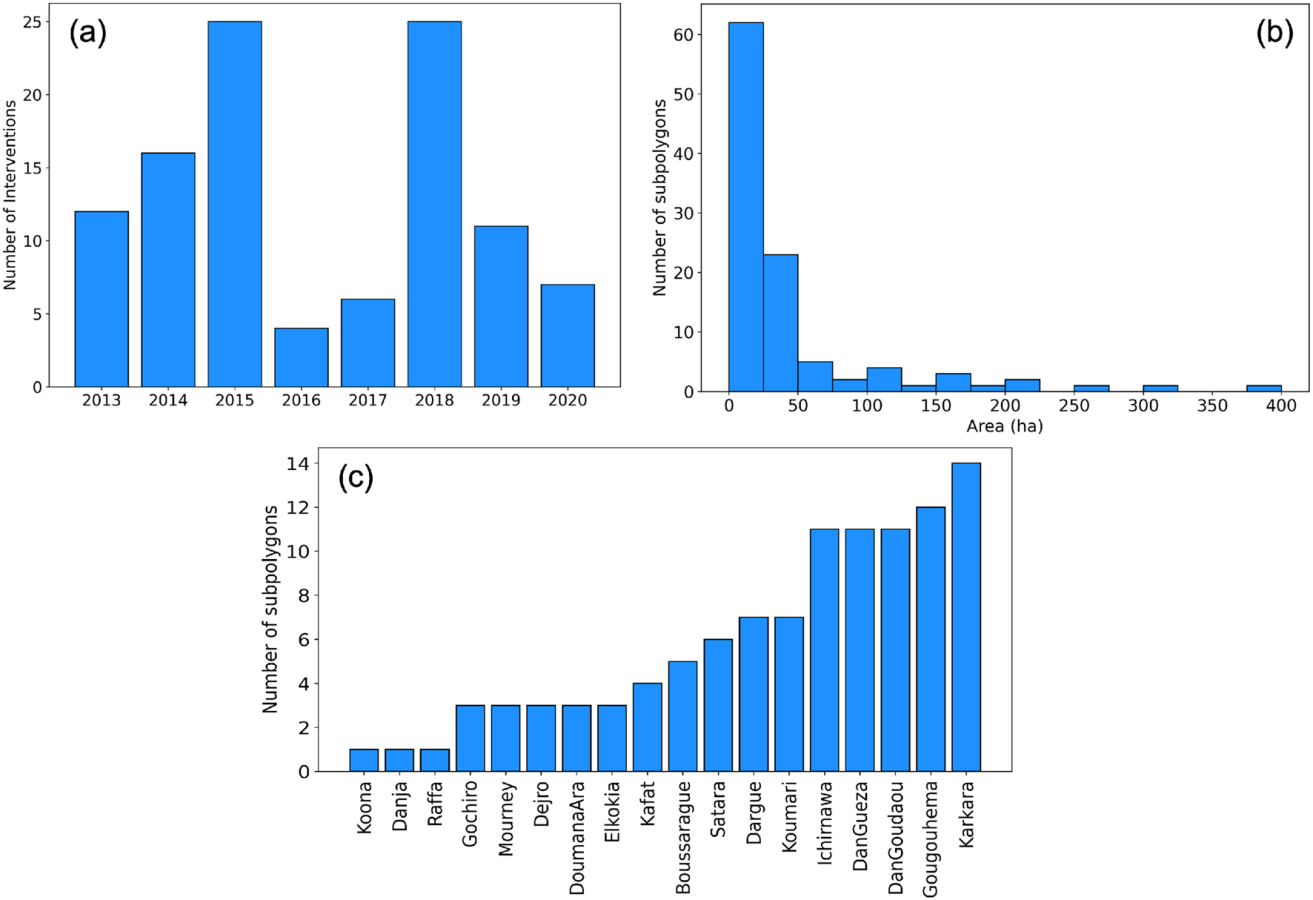
(**a**) shows the number of polygons intervened every year between 2013 and 2020. (**b**) is the histogram of the area in hectares for each of the polygons showing the distribution of polygon sizes. (**c**) elatively few were intervene represents the number of polygons at each of the sites that were intervened during the study period in the region.

WFP is working on more than 300 sites for SWC interventions in southern Niger and has recorded specific geographic outlines of half-moons at 18 sites (Fig. 7) from four regions - Maradi, Zinder, Tahoua, and Tillaberi. Each site has several areas in which half-moons are concentrated (termed as intervention polygons hereon) that were either developed as pastoral or agricultural half-moons (Fig. 7). Some of the sites had only one polygon (e.g. Koona, Raffa and Danja) whereas Karkara had 14 polygons (Fig. 8c). However, it should be noted that not all of the polygons from a given site were intervened simultaneously. Most polygons were intervened during 2015 (24) and 2018 (26), while relatively few were intervened in 2016 (4) and 2017 (6) (Fig. 8a). These 18 sites include 101 intervention polygons and cover approximately 4400 ha of the total area developed with an average of nearly 42 ha per site. Only 14 sites had an area greater than 100 ha while 62 sites covered less than 25 ha of area. Figure 8b shows the area distribution for all the sites.

**Figure 9:**
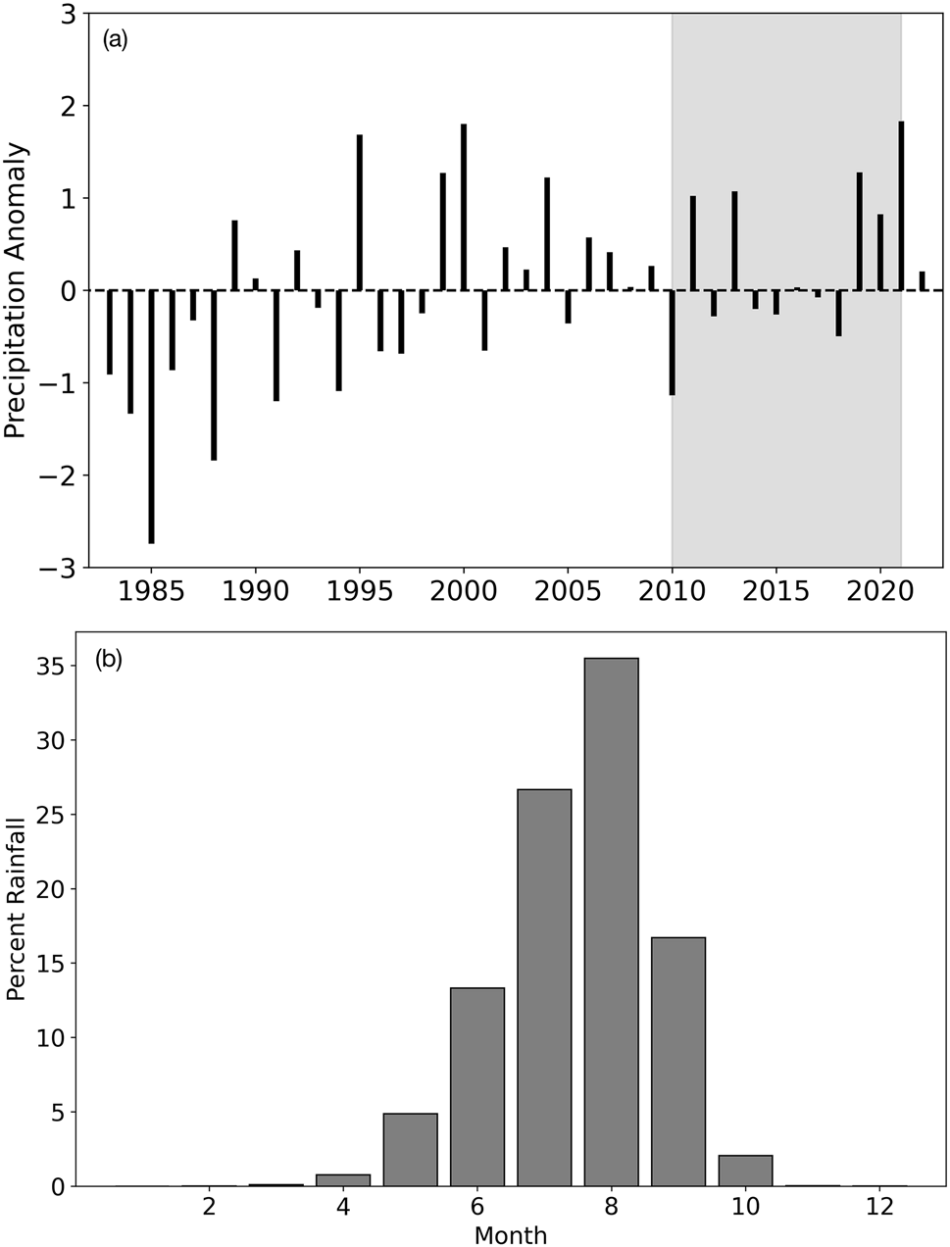
(**a**) standard rainfall anomaly for southern Niger over the last 40 years (1982–2021). The shaded portion highlights the time period analyzed in this study. (**b**) shows the percent monthly distribution of long-term rainfall in the region, indicating a clear distinct rainy season and long summer months.

Most of the fields in southern Niger are rainfed making farmers vulnerable to climate conditions and variable rainfall patterns. On average, in the last 40 years (1982–2021) southwestern Niger has experienced nearly 450 mm of rainfall annually. However, total rainfall can vary significantly annually (standard deviation of 70 mm, approx.) with as low as 270 mm (1984) to more than 630 mm (2020) in a single year. Figure 9a shows standardized anomalies of annual rainfall in southern Niger. In general, rainfall has been either normal (less than ± 0.5 deviations from normal) or above average. In particular, the years 2018–020 showed significantly higher than normal rainfall in the region. Furthermore, the region has a very distinct rainy season (Jun–Sept) when more than 90% of the total annual rainfall is observed (Fig. 9b). Therefore, the SWC measures in the region can provide much-needed moisture availability for longer duration.

### Satellite data

With a repeat cycle of 16 days and moderately high spatial resolution (30 m), the historical record of Landsat data extends to the 1980s. Several vegetation indexes have been developed to quantify vegetation health, including the NDVI, Enhanced Vegetation Index (EVI^63^), and Soil Adjusted Vegetation Index (SAVI^64^) etc. Vegetation changes are slow (spanning over several days) and Landsat’s 16-day revisit frequency can capture the natural vegetative cycle. However, frequent cloud cover poses a challenge to the use of visible and near-infrared band-based indexes such as NDVI. That is particularly true for data collected before 2010 when relatively fewer satellite overpasses are available for analysis. In this study, we used Landsat 7 data from Google Earth Engine image collection after accounting for quality flags, clouds, and cloud shadows^65^. Moreover, the rainfall data for contextual assessment was available from Climate Hazards Infrared Precipitation with Station Data (CHIRPS^66^). CHIRPS is a satellite-driven rainfall product that has been corrected using ground observations from across the globe. A 5 day (pentad) CHIRPS product (1982–2021) at 5 km spatial resolution was used in this study. Gridded CHRIPS precipitation data has been extensively evaluated and applied across the globe^66–69^.

### Temporal analysis

Although, the study period includes the year 2020, any site that were developed in the year 2020 were not included in this analysis due to limited post-intervention sample size. Therefore, out of 108 possible polygons from 18 sites, only 101 were examined in this study. The average size of the polygons is approximately 42 ha, equivalent to about 450 Landsat pixels. Cloud and cloud shadow masked Landsat imageries were used to compute NDVI for each pixel within each polygon and then aggregated over the polygon for temporal analysis. In this study, the NDVI values were analyzed at monthly and annual scales. The annual peak (95^th^) percentile data were analyzed to assess the variability in polygons’ average NDVI values over the years. Since this study was designed retrospectively, we could not randomly assign matching control sites. It is possible that despite being from close proximity and similar agro-ecological classifications, the physical characteristics could vary, thereby influencing the results. To address this challenge, we leveraged the longitudinal characteristics of the Landsat record to establish a retrospective “baseline” using the preceding years of the sites that were developed at a later stage (after 2017) as controls for the treatment sites that were developed between 2013-2015 from the same region.

### Control analysis

During the planning and implementation of these interventions, no control site was specifically identified. Therefore, for this analysis, we have taken an indirect approach where a pair of polygons from the same site that were developed a few years apart were used as an experiment (or intervened) and control sites. This approach ensured that the later-intervened area was suitable for the intervention, and hence is an appropriate comparator. We applied three criteria for selecting the control and intervention pairs by ensuring:

- that the polygons were nearby (as part of the same larger site)
- that the sites are from the same livelihood zones (agricultural or pastoral)
- that the polygons have interventions at least 3 years apart.

The first criterion ensures that both sites have similar weather patterns and cropping/grazing practices. The second ensures the similarity in vegetation types and patterns, and the final criterion ensures that the evaluation can focus on those years where the intervention sites are expected to increase in vegetation, whereas the control sites are anticipated to follow the pre-intervention vegetation patterns. Effectively, the site that was developed earlier becomes the intervention site or experimental site, whereas a site with a later intervention date can be used as control site until an intervention happens at that polygon. Using this approach, a total of 7 pairs (Table 2) across multiple sites were found that can be used for control analysis. The duration of control years ranged from 3 to 6 depending upon the site and intervention years. The minimum area selected in either control or intervention was 13 ha, (150 Landsat pixels) to ensure a statistically substantial pixel count for estimating NDVI. We divided NDVI data into two time periods:

- Baseline period – Years 2010 to the intervention year on experimental sites, pre-intervention baseline years.
- Experiment period – From intervention on the experimental site to the intervention on the control site.

For example, polygon 1 at Dan Goudau site was developed in 2013, whereas polygon 0 was developed in 2018. Therefore, polygon 1 becomes an experimental polygon whereas polygon 0 can be used as a control until 2018. The years 2010–2013 are termed as the baseline period; 2014–2018 as the experiment period; and 2019–2020 as the post-experiment years, which are excluded from this current analysis, due to the short length of time available to make any statistical inferences.

## Data Availability

Model codes and sample data are publicly available at https://github.com/Vikalp86/Resilience_Mapping. Further data/analysis used in this study also available from the corresponding author on reasonable request.
